# Supplementary material for: Dynamic transcriptomic profiles of zebrafish gills in response to zinc supplementation
Source: BMC Genomics. 2010 Oct 11;11:553. doi: 10.1186/1471-2164-11-553 (PMC3091702; doi:10.1186/1471-2164-11-553)
Supplement: Additional file 2 — Interactive Direct Interaction Network representing the molecular interactions between zinc, copper, iron, calcium and proteins encoded by transcripts changed by zinc supplementation. Mini web-site containing index.html and hyperlinked pages in subdirectory describing a Direct Interaction Network automatically generated based on curated interactions contained within the proprietary PathwayArchitect database. Ovals represent proteins and the circles symbolize metal ions. Objects are coloured by their abundance in zebrafish at the time-point they were significantly different from the control is a scale from -4 fold (dark green) to +4 fold (dark red). Where significant differences were found at more than one time-point, the colour overlay shows expression at the first instance. Dark blue squares denote 'binding', and light blue squares 'expression'; green squares stand for 'regulation', green diamonds for 'metabolism', and green circles for 'promoter binding'. Arrow heads indicate directionality of the interaction where annotated. All nodes and edges can be further interrogated by selecting the relative area of the image. [file 1471-2164-11-553-S2.zip › PathwayArchitect Zn xs DIN/505176.html]

# TRANSPORT:

|  |  |
| --- | --- |
| Type | TRANSPORT |
| Effect | None |


---

|  |  |
| --- | --- |
| Score | 0 |


---

|  |  |
| --- | --- |
| Reference Count | 25 |


---

|  |  |
| --- | --- |
| Mechanism | Unknown |


---

|  |  |
| --- | --- |
| Reference:0 || Sentence | "Two iron transporters, divalent metal transporter1 (DMT1) and ferroportin1 (FPN1) have been identified; however, their role during infancy is unknown." |
| PMID | 12958019 |
| Year | 2003 |
| Species | Rat |
| Journal | Am J Physiol Gastrointest Liver Physiol |
| RefScore | 1 |
| Source | PArchNLP |
  |
|  |  |


---

|  |  |
| --- | --- |
 Reference:1 || Sentence | "Iron-regulated transporter protein 1 (IREG1 or ferroportin 1) is a transmembrane iron transporter that has been described in macrophages and hepatocytes." |
| PMID | 12972034 |
| Year | 2003 |
| Species | Human |
| Journal | Blood Cells Mol Dis |
| RefScore | 1 |
| Source | PArchNLP |
  ||  |  |


---

|  |  |
| --- | --- |
 Reference:2 || Sentence | "The recent identification of other proteins of iron and copper metabolism, for example, copper ATPases and the membrane iron transporters DCT1/DMT1/Nramp2 and IREG1/MTP1/ferroportinl, are likely to fill crucial pathway gaps." |
| PMID | 12572662 |
| Year | 2003 |
| Species | Human |
| Journal | Biometals |
| RefScore | 1 |
| Source | PArchNLP |
  ||  |  |


---

|  |  |
| --- | --- |
 Reference:3 || Sentence | "Northern blot analysis revealed that expression of the apical iron transporter divalent metal transporter-1 (DMT1), the basolateral transporter ferroportin-1 (Fpn1), and the putative ferroxidase hephaestin (Heph) was upregulated by copper supplementation, whereas the recently identified ferrireductase duodenal cytochrome b (Dcytb) was not." |
| PMID | 11842003 |
| Year | 2002 |
| Species | Human |
| Journal | Am J Physiol Gastrointest Liver Physiol |
| RefScore | 1 |
| Source | PArchNLP |
  ||  |  |


---

|  |  |
| --- | --- |
 Reference:4 || Sentence | "The characterisation of specific iron transporters such as the divalent metallic transporter and ferroportin may hold promise for the development of 'smart' compounds capable of blocking iron transport." |
| PMID | 14640923 |
| Year | 2003 |
| Species | Human |
| Journal | Expert Opin Pharmacother |
| RefScore | 1 |
| Source | PArchNLP |
  ||  |  |


---

|  |  |
| --- | --- |
 Reference:5 || Sentence | "Ferroportin1 is a newly discovered molecule that may play a role in iron export." |
| PMID | 11809412 |
| Year | 2002 |
| Species | Human |
| Journal | Int J Biochem Cell Biol |
| RefScore | 0 |
| Source | PArchNLP |
  ||  |  |


---

|  |  |
| --- | --- |
 Reference:6 || Sentence | "Recently, a number of novel genes involved in iron metabolism, such as the iron uptake transporter DMT1/DCT1/Nramp2 and the iron export transporter IREG1/ferroportin1/MTP1, have been identified, providing important insights about molecular aspects of intestinal iron absorption and its regulation." |
| PMID | 11925462 |
| Year | 2002 |
| Species | Human |
| Journal | J Nutr |
| RefScore | 2 |
| Source | PArchNLP |
  ||  |  |


---

|  |  |
| --- | --- |
 Reference:7 || Sentence | "The possibility that hepcidin regulates the expression of ferroportin 1 (FPT1), the basolateral iron transporter, was examined in rats after administration of LPS, an iron chelator, or His-tagged recombinant hepcidin (His-rHepc)." |
| PMID | 14592944 |
| Year | 2003 |
| Species | Rat |
| Journal | Am J Physiol Gastrointest Liver Physiol |
| RefScore | 1 |
| Source | PArchNLP |
  ||  |  |


---

|  |  |
| --- | --- |
 Reference:8 || Sentence | "Two intestinal iron transporters, divalent metal transporter 1 (DMT1) and ferroportin 1 (FPN1), were recently identified." |
| PMID | 14668284 |
| Year | 2003 |
| Species | Human |
|  | Rat |
| Journal | Am J Clin Nutr |
| RefScore | 1 |
| Source | PArchNLP |
  ||  |  |


---

|  |  |
| --- | --- |
 Reference:9 || Sentence | "CONCLUSIONS: DMT1 and FP1 are centrally involved in iron uptake/transfer in the duodenum and in the adaptive changes of iron homeostasis to iron deficiency and overload." |
| PMID | 11313311 |
| Year | 2001 |
| Species | Human |
| Journal | Gastroenterology |
| RefScore | 2 |
| Source | PArchNLP |
  ||  |  |


---

|  |  |
| --- | --- |
 Reference:10 || Sentence | "The newly described iron transporter, ferroportin (MTP1, IREG1), is expressed in a variety of tissues including the duodenum and cells of the mononuclear phagocyte system (MPS)." |
| PMID | 12547222 |
| Year | 2002 |
| Species | Human |
| Journal | Blood Cells Mol Dis |
| RefScore | 1 |
| Source | PArchNLP |
  ||  |  |


---

|  |  |
| --- | --- |
 Reference:11 || Sentence | "Previous reports concerning gene expression levels of the duodenal iron transporters DMT1 and IREG1 in HH patients and animal models are controversial, however, and in many cases only mRNA expression levels were investigated." |
| PMID | 14618243 |
| Year | 2003 |
| Species | Mouse |
|  | Human |
| Journal | J Mol Med |
| RefScore | 1 |
| Source | PArchNLP |
  ||  |  |


---

|  |  |
| --- | --- |
 Reference:12 || Sentence | "HFE deficiency in intestinal crypt cells is thought to cause intestinal iron deficiency and greater expression of iron transporters such as SLC11A2 (also called DMT1, DCT1 and NRAMP2) and SLC11A3 (also called IREG1, ferroportin and MTP1; ref. 3)." |
| PMID | 12704390 |
| Year | 2003 |
| Species | Mouse |
|  | Human |
| Journal | Nat Genet |
| RefScore | 1 |
| Source | PArchNLP |
  ||  |  |


---

|  |  |
| --- | --- |
 Reference:13 || Sentence | "Zebrafish ferroportin1 is required for the transport of iron from maternally derived yolk stores to the circulation and functions as an iron exporter when expressed in Xenopus oocytes." |
| PMID | 10693807 |
| Year | 2000 |
| Species | Mouse |
|  | Human |
| Journal | Nature |
| RefScore | 1 |
| Source | PArchNLP |
  ||  |  |


---

|  |  |
| --- | --- |
 Reference:14 || Sentence | "Mammalian Ferroportin1 is expressed at the basolateral surface of duodenal enterocytes and could export cellular iron into the circulation." |
| PMID | 10693807 |
| Year | 2000 |
| Species | Mouse |
|  | Human |
| Journal | Nature |
| RefScore | 1 |
| Source | PArchNLP |
  ||  |  |


---

|  |  |
| --- | --- |
 Reference:15 || Sentence | "Ireg1 (SLC11A3, also known as Ferroportin1 or Mtp1) is the putative intestinal basolateral iron transporter." |
| PMID | 12730111 |
| Year | 2003 |
| Species | Mouse |
| Journal | Blood |
| RefScore | 1 |
| Source | PArchNLP |
  ||  |  |


---

|  |  |
| --- | --- |
 Reference:16 || Sentence | "BACKGROUND & AIMS: An increased duodenal expression of the iron transporters, divalent-metal-transporter-1, and ferroportin is observed in patients with iron deficiency or hereditary hemochromatosis." |
| PMID | 12949720 |
| Year | 2003 |
| Species | Human |
| Journal | Gastroenterology |
| RefScore | 2 |
| Source | PArchNLP |
  ||  |  |


---

|  |  |
| --- | --- |
 Reference:17 || Sentence | "RESULTS: In iron deficiency a coordinated upregulation of the iron transporters divalent-metal-transporter-1 and ferroportin and of duodenal-cytochrome b and hephaestin was found, whereas in patients with HFE and non-HFE-associated hemochromatosis duodenal-cytochrome b and hephaestin protein and mRNA expression were not significantly different from control subjects." |
| PMID | 12949720 |
| Year | 2003 |
| Species | Human |
| Journal | Gastroenterology |
| RefScore | 2 |
| Source | PArchNLP |
  ||  |  |


---

|  |  |
| --- | --- |
 Reference:18 || Sentence | "We suggest that hephaestin, by way of its ferroxidase activity, facilitates iron export from intestinal enterocytes, most likely in cooperation with the basolateral iron transporter, Ireg1." |
| PMID | 14751926 |
| Year | 2004 |
| Species | Mouse |
| Journal | Blood |
| RefScore | 1 |
| Source | PArchNLP |
  ||  |  |


---

|  |  |
| --- | --- |
 Reference:19 || Sentence | TNFalpha mediated an early induction in both iron import and iron export, which were associated with increased DMT-1 and IREG-1 mRNA and protein expression (P<0.05). |
| Year | 2005 |
| PMID | 15901240 |
| Species | Human |
| Journal | Biochem J |
| RefScore | 1 |
| Source | PArchNLP |
  ||  |  |


---

|  |  |
| --- | --- |
 Reference:20 || Sentence | Release of iron from the enterocyte into the plasma involves the iron-regulated transporter-1/metal transporter protein-1 (IREG-1/MTP-1, ferroportin) but ferroportin is also found on the apical membrane. |
| Year | 2004 |
| PMID | 15114483 |
| Species | Rat |
| Journal | Pflugers Arch |
| RefScore | 1 |
| Source | PArchNLP |
  ||  |  |


---

|  |  |
| --- | --- |
 Reference:21 || Sentence | Whether intestinal iron transporters, divalent metal transporter 1 (DMT1) and ferroportin 1 (FPN1), are present in the mammary gland and are involved in iron transfer into milk are unknown. |
| PMID | 15699234 |
| Year | 2005 |
| Species | Rat |
| Journal | Am J Clin Nutr |
| RefScore | 1 |
| Source | PArchNLP |
  ||  |  |


---

|  |  |
| --- | --- |
 Reference:22 || Sentence | CONCLUSIONS: The results show that DMT1 and FPN1 concentrations are higher during early lactation and are possibly involved in iron transfer into milk. |
| PMID | 15699234 |
| Year | 2005 |
| Species | Rat |
| Journal | Am J Clin Nutr |
| RefScore | 1 |
| Source | PArchNLP |
  ||  |  |


---

|  |  |
| --- | --- |
 Reference:23 || Sentence | The surviving cells evoked an adaptative response consisting of increased synthesis of the iron-storage protein ferritin and the iron export transporter IREG1, and decreased synthesis of the iron import transporter DMT1. |
| PMID | 15667655 |
| Year | 2005 |
| Species | Mouse |
|  | Human |
|  | Rat |
| Journal | BMC Neurosci |
| RefScore | 2 |
| Source | PArchNLP |
  ||  |  |


---

|  |  |
| --- | --- |
 Reference:24 || Sentence | Several iron transporters and regulators were recently characterized, including DMT1 and ferroportin/Ireg1 that transport iron through membranes, and HFE that regulates TfR-mediated iron uptake. |
| PMID | 15880641 |
| Year | 2005 |
| Species | Human |
| Journal | J Cell Physiol |
| RefScore | 1 |
| Source | PArchNLP |
  |


---

|  |  |
| --- | --- |
